# An Experimental and Numerical Study of Polyelectrolyte Hydrogel Ionic Diodes: Towards Electrical Detection of Charged Biomolecules

**DOI:** 10.3390/s21248279

**Published:** 2021-12-10

**Authors:** Chenwei Xiong, Boyin Zhang, Rong Zhang, Yifan Liu

**Affiliations:** Division of Chemistry and Physical Biology, School of Physical Science and Technology, ShanghaiTech University, Shanghai 201210, China; xiongcw@shanghaitech.edu.cn (C.X.); zhangby2@shanghaitech.edu.cn (B.Z.); zhangrong1@shanghaitech.edu.cn (R.Z.)

**Keywords:** microfluidics, ion transport, polyelectrolyte hydrogel, ionic diode, biosensor

## Abstract

Polyelectrolyte hydrogel ionic diodes (PHIDs) have recently emerged as a unique set of iontronic devices. Such diodes are built on microfluidic chips that feature polyelectrolyte hydrogel junctions and rectify ionic currents owing to the heterogeneous distribution and transport of ions across the junctions. In this paper, we provide the first account of a study on the ion transport behavior of PHIDs through an experimental investigation and numerical simulation. The effects of bulk ionic strength and hydrogel pore confinement are experimentally investigated. The ionic current rectification (ICR) exhibits saturation in a micromolar regime and responds to hydrogel pore size, which is subsequently verified in a simulation. Furthermore, we experimentally show that the rectification is sensitive to the dose of immobilized DNA with an exhibited sensitivity of 1 ng/μL. We anticipate our findings would be beneficial to the design of PHID-based biosensors for electrical detection of charged biomolecules.

## 1. Introduction

In recent years, hydrogel-based materials have been broadly used in tissue engineering [1], translational medicine [2], and biosensing [3,4] for their biocompatibility, plasticity, and semi-permeability [5]. Among various classes of hydrogels, polyelectrolyte hydrogel has gained particular interest in the field of biomedical iontronic devices due to its exhibited ion conductivity and selectivity [6]. With such a unique type of material, scientists have developed an array of functional iontronic devices such as ionic diodes [7,8] and transistors [9], and they have demonstrated their potential in ion amplification [10] and all-ionic circuits [11].

**Figure 1:**
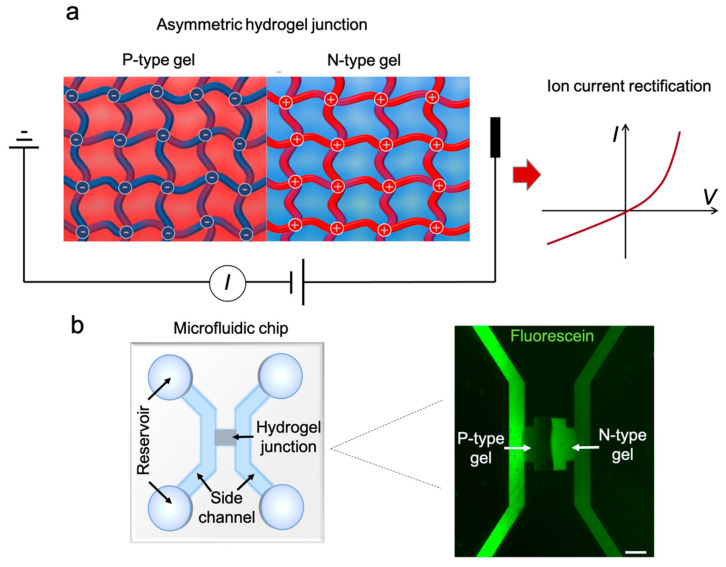
Illustration of a PHID device and its working principle: (**a**) Conceptual illustration of a polyelectrolyte heterojunction. The intertwined meshes represent hydrogel backbones with their color denoting charge polarities (red, positively charged and blue, negatively charged). The colored background denotes the counter ions in the nanopores (red, positively charged ions and blue, negatively charged ions). The asymmetry in distribution of charge carriers leads to asymmetric I-V characteristics; (**b**) the PHID device layout (left) and a fluorescent micrography showing hydrogel heterojunction of a representative device. Scale bar, 50 μm.

Polyelectrolyte hydrogel ionic diodes are structurally analogous to semiconductor diodes [12]. Figure 1a depicts a representative schematic of a PHID device, which is an ionic PN junction consisting of two segments of oppositely charged hydrogels. The polyelectrolyte backbone of P-type gel exhibits a strong negative charge, and thus repels co-ions within its surrounding electrical double layers. Therefore, P-type gel selectively allows the transport of positively charged counter-ions, and vice versa, N-type gel is permeable preferentially to negative charge carriers. When an external electrical field is applied to this ionic heterojunction, it rectifies the ionic current depending on the bias polarity, leading to an asymmetric current-voltage (I-V) response. The ionic rectification mechanism of PHIDs is considered to be identical to their nanoscale counterpart, i.e., nanofluidic diodes [13].

In nanofluidic diodes, ionic rectification takes place in a fluidic nanochannel where the critical dimension is comparable to the Debye length, and thus ion distribution is dominated by the surface charge rather than the bulk ionic strength. The rectification also demands heterogeneity across the channel which can be asymmetric surface charge distribution or channel geometry profile. In both PHIDs and nanofluidic diodes, the rectification behavior can be explained by the enrichment and depletion of charge carriers across the structures in response to distinct bias polarities [13]. In regard to nanofluidic diodes, several quantitative models have been established to study their ICR [14,15]. Notably, Kubeil et al. performed numerical simulations based on Poisson–Nernst–Planck (PNP) equations to investigate the geometric effect on ICR in conical nanopores, and found that ICR could be increased by decreasing the half-cone angle [14]. With these theoretical efforts, novel nanofluidic diodes have been experimentally developed to achieve various chemical and biomedical applications [16]. We previously demonstrated label-free quantification of nucleic acids in a polymerase chain reaction (PCR) using functionalized glass nanopipettes [17], and a nanoslit fluidic diode microchip for the detection of human cardiac troponin T (cTnT) at femtomolar levels [18].

Compared to nanofluidic diodes, PHIDs offer a couple of unique advantages. First, the fabrication of nanofluidic diodes often requires expensive and cumbersome advanced manufacturing techniques (e.g., E-beam lithography and focused ion beam lithography [19]), which limits their widespread applications. On the contrary, in regard to PHIDs, polyelectrolyte hydrogels are inexpensive and can be precisely patterned using standard UV (365 nm) photolithography [20]. For instance, Lim et al. developed an open-junction ionic diode based on microfluidic chips and UV photopolymerization, and demonstrated unique ion sensing and amplification mechanisms [10]. Second, hydrogels are nanoporous and stretchable in nature, which is ideal for the development of flexible iontronic devices [21]. Lee et al. reported on the fabrication of a stretchable PHID with co-polyelectrolyte hydrogels modified with methacrylated polysaccharides in which the device exhibited continuous rectification over hundreds of cycles of stretching [7]. These advantages render PHIDs extremely suitable for novel biosensing platforms. However, the ion transport characteristics of PHIDs has not yet been well studied, either experimentally or numerically [22,23]. In this study, we design and fabricate hydrogel heterojunctions on microfluidic chips to experimentally study their ion transport characteristics. We also perform numerical simulations to model the ion transport process, and study the effects of bulk ionic strength, hydrogel pore size, and surface charge density on ICR performance. Furthermore, based on experimental and numerical means, we investigate whether a PHID device can be applied to electrical detection of charged biomolecules.

## 2. Materials and Methods

### 2.1. Device Fabrication

The PHID was realized by creating hydrogel PN junctions on microfluidic chips. The chips were fabricated through standard polydimethylsiloxane (PDMS)-based soft lithography. To start with, microfluidic channels were designed in AutoCAD and printed as a plastic film photomask. Then, the patterns on the mask were transferred onto a 3 inch silicon wafer through photolithography. The lithography began with spin-coating (2200 rpm) a layer of SU8-3025 photoresist (Microchem) on the wafer. Then, UV exposure (200 s under a Thorlabs COP1-A UV light) and developing steps were applied. A PDMS precursor mixture (Dow Corning SYLGARD 184 Silicone Elastomer) was poured on the patterned silicon mold and cured at 60 °C overnight. The cured PDMS slab was peeled off from the mold and cut into individual chips. Inlet and outlet ports were created with a custom-made hole puncher. The chips were finally sealed by bonding to 100 μm thick glass slides.

Polyelectrolyte hydrogel junctions were formed on as-fabricated microfluidic chips through a two-step photocuring process. First, the microfluidic channels were treated with the mixed solution of 3-(trimethoxysilyl)propyl methacrylate (TMSMA), methanol, and acetic acid (weight ratio of 1:8.5:0.5) for 5 min, and then washed with methanol. Next, the channels were filled with a P-type polyelectrolyte hydrogel pre-solution, and then subjected to photocuring (Dymax 38465) for 10 s. During the curing process, the chip was covered with a chromium-glass dark mask, only exposing a 100 μm wide region at the channel junction. After a thorough washing with 10 mM KCl solution, the photocuring process was repeated with a N-type polyelectrolyte hydrogel pre-solution. The 1X hydrogel pre-solution consisted of 20 wt% monomer (P-type, 3-sulfopropyl acrylate potassium salt and N-type, diallyl-dimethylammonium chloride), 1 wt% crosslinker (*N*,*N*′-methylenebisacrylamide), and 0.2 wt% photo-initiator (lithium phenyl-2,4,6-trimethylbenzoylphosphinate) in water. The 1X concentration was used in all the experiments, except as specifically stated. Finally, the formed hydrogel PN junction was incubated in 10 mM KCl overnight before testing.

### 2.2. Electrical Measurement

The electrical measurements of the PHID were performed on an electrochemical workstation (CH Instruments). To record the I-V behavior of the hydrogel PN junction, two Ag/AgCl wire electrodes were inserted into corresponding reservoirs of the microfluidic chip. The potential bias between the two electrodes was scanned from −4 to 4 V at a rate of 4 V/s. Unless otherwise stated, the electrolyte condition was 10 mM KCl in all the experiments.

### 2.3. Microscopic Imaging

The fluorescent micrograph of hydrogel heterojunction was obtained under an Olympus inverted fluorescent microscope (Eclipse Ti2). The scanning electron microscopic (SEM) image was obtained using a JEOL JSM-IT500 microscope. Before imaging, the hydrogel samples were freeze-dried in a critical point dryer (SAMDRI-795, Tousimis, Rockville, MD, USA) and subsequently coated with 5 nm gold.

### 2.4. DNA Detection

A 500 base pairs (bp) DNA fragment of *Escherichia coli* was PCR amplified using a forward primer GAATACCCCGATTGGTGATG and a reverse primer ATTCTCACCGGATTCAGTCG. The PCR product was purified with a PCR clean-up kit (Zymo Research) and diluted to the desired concentration. In the detection experiments, the DNA solution was loaded into the side channel facing the N-type gel and incubated for 5 min. Then, the channel was thoroughly washed with 10 mM KCl solution before I-V testing.

### 2.5. Numerical Procedure

The numerical study used PNP equation system to describe the ion transport in a polyelectrolyte hydrogel PN junction:

(1) $$\nabla^{2}\phi =-\frac{F}{\varepsilon }\underset{i}{\sum }z_{i}c_{i}$$

(2) $$J_{i}=-D_{i}\nabla c_{i}-\frac{z_{i}F}{\dot{R}T}D_{i}c_{i}\nabla \phi$$

where $J_{i}$ is the ionic flux, $D_{i}$ is the diffusion coefficient, $c_{i}$ is the concentration, $z_{i}$ is the charge of the species i, ϕ is the electrical potential, and ε is the dielectric constant of the fluid medium. F and $\dot{R}$ denote the Faraday constant and the ideal gas constant, respectively. As previously reported, the Navier–Stokes equations, which describe the motion of fluids, can be neglected in this model [14].

For the ionic carriers, only two types of ions, K^+^ and Cl^−^, were considered in the model, with $D\;\left(K^{+}\right)=1.957\times 10^{9}\;m^{2}/s$ and $D\;\left(Cl^{-}\right)=2.032\times 10^{9}\;m^{2}/s$. Their concentrations $c_{i}$ varied from 1 to 500 μM. The Debye length, $\lambda_{D}$, can be derived as:

(3) $$\lambda_{D}=\sqrt{\frac{\varepsilon k_{B}T}{2n_{0}e^{2}}}$$

where $k_{B}$ is the Boltzmann constant, T is the absolute temperature, $n_{0}$ is the number density of the ions, and e is the elementary charge.

The numerical procedure was conducted under COMSOL Multiphysics version 5.6 based on the finite element method. By leveraging the axial symmetry of the polyelectrolyte hydrogel heterojunction, the problem was reduced to a 2D axial symmetric computational domain (Figure S1). The entire structure is symmetric along the center axis. The nanoporous hydrogel junction was modeled as a 2D nanopillar matrix, which was sandwiched by two reservoirs filled with KCl electrolyte solution. The junction was divided into two equal sections. In the junction, the nanopillars represent charged hydrogel backbones, whereas gaps between two adjacent pillars refer to nanopores filled with electrolyte solution. In the procedure, the nanopore size (w) was set to 10–40 nm. The surface charge density of the pillars was set as ±1–3 mC/m^2^ for N-gel (positive surface charge) and P gel (negative surface charge) sections. The rectangular heterojunction had a length of 900 nm and a width of 270 nm. The potential applied was varied from −1 to +1 V against the ground potential. The size of the two reservoirs was chosen to be 1 × 1 μm^2^ to eliminate the influence from reservoir walls. In all the simulations, the mesh size was set in the range of 0.1–0.5 nm to fully resolve the ionic fluxes and obtain accurate results. All the boundary condition settings used in the computational domain are detailed in Table S1.

## 3. Results and Discussion

### 3.1. Experimental Characterization of the PHID Device

The schematic of the complete PHID device is shown in Figure 1b (left). The device consists of two side channels bridged by a hydrogel PN junction. The fluorescent micrograph (Figure 1b, right) highlights the junction area. To better visualize the hydrogel, the device was filled with 1 mM fluorescein solution. Because fluorescein molecules are negatively charged, they accumulate at the positively charged N-type gel region, rendering it in bright. On the contrary, the negatively charged P-type gel repels fluorescein and is thus shown in dark. As depicted in the micrograph, the two hydrogel blocks are around 100 μm wide and adjacent to each other without noticeable gaps in between. The two side channels are connected solely via the hydrogel PN junction, evading any potential leaking currents that may cause inferences.

**Figure 2:**
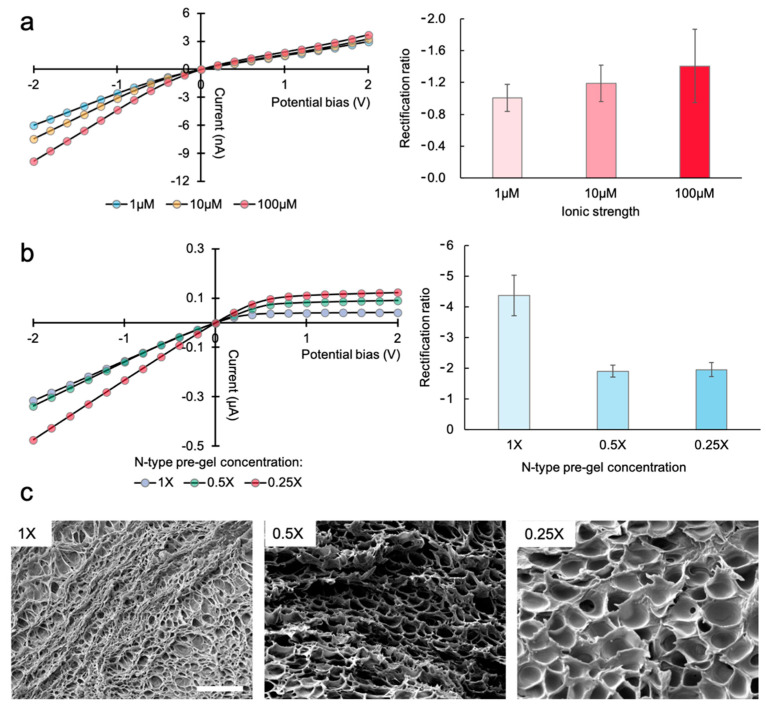
Characterization of the representative PHID device: (**a**) I-V curves (left) and rectification levels (right, n = 3) of a representative device obtained at various ionic strengths; (**b**) I-V curves (left) and rectification levels (right, n = 3) obtained at different N-type gel concentrations. The electrolyte solution is kept to 10 mM KCl and P-type gel concentration is kept to 1X; (**c**) SEM images of dried N-type gel blocks at different concentrations. Scale bar, 1 μm.

The PHID device, in this study, rectifies ion currents due to electrostatic field effect; charged hydrogel backbones attract counterions in the electrolyte solution within the electrical double layers, which lead to a lopsided distribution of charge carriers (positive and negative ions) in either type of PE gel. Therefore, the ICR behavior should be sensitive to the bulk electrolyte strength, which determines the characteristic length of electrical double layers. For other types of ionic diodes, i.e., 70 nm conical nanochannels, the rectification level is reversely correlated with the ionic strength until saturation is reached (at micromolar scale). For the PHID, however, we expect the trend to be different, as the hydrogels ought to contain more confined and irregular nanopores. In fact, the relation between the rectification level and ionic strength for PHIDs has not been explored. Therefore, to evaluate this, first, we monitored the I-V response of a representative PHID device at micromolar ionic strengths, Figure 2a. Here, we define the logarithmic rectification ratio (*R*), i.e., $R=log_{2}\left(I_{+max}/I_{-max}\right)$, where $I_{+max}$ and $I_{-max}$ refer to ionic current at maximum positive and negative biases, respectively. The same definition has also been used in previous reports to quantify the ICR of nanofluidic diodes [17,18]. As seen, only a slight change in *R*, i.e., 0.4, is observed from 1 to 100 μM, indicating that the rectification reached saturation at a micromolar scale. The results suggest that unlike other ionic diodes the ionic behavior of PHIDs should be sensitive to higher ionic strength, for example, in the range of millimolar, possibly owing to more confined geometry and enhanced electrostatic field effect. Indeed, we found a higher rectification in a later experiment (Figure 2b, 1X) in which the ionic strength was elevated to 10 mM with other conditions unchanged.

Next, we investigated whether tuning the hydrogel confinement level would affect the ionic rectification behavior. In this experiment, we lowered the original (1X) concentration of the N-type gel precursor solution. The SEM images in Figure 2c show that the mesh size of N-type gel is significantly enlarged when diluting the precursor solution. The average pore size is 0.23, 1.47, and 2.96 μm for 1X, 0.5X, and 0.25X N-type gels, respectively (n = 15). Here, it is worthwhile noting that the measured pore sizes may not represent the actual pore sizes of the hydrogel, as the pores might experience swelling during the sample preparation procedure for SEM. The plots in Figure 2b clearly show that the highest rectification level, i.e., R = −4.4, is obtained with 1X N-type gel which possesses the highest confinement. These results confirm the ideal working condition for a PHID device to obtain a reasonably high and sensitive rectification.

### 3.2. Numerical Study of the Ion Transport Behavior of PHID

**Figure 3:**
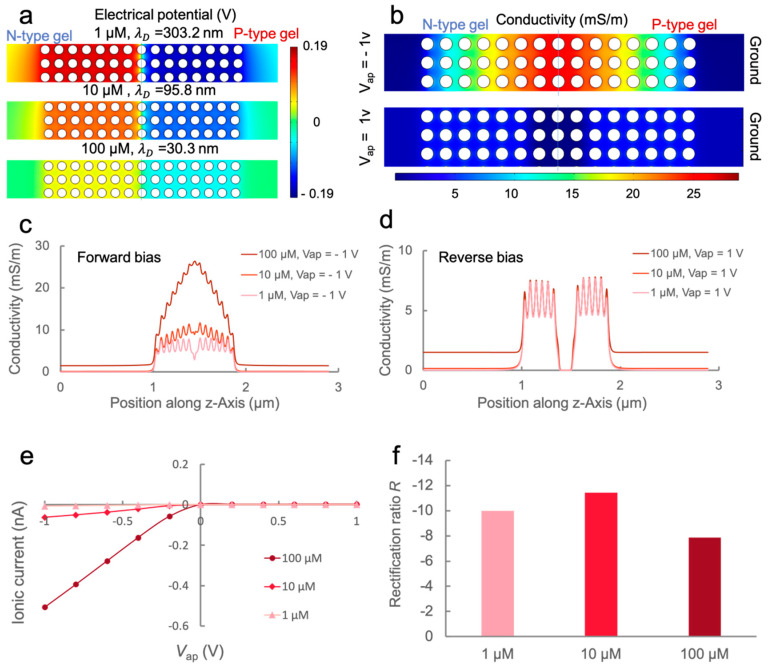
Simulation of the effect of ionic strength on ion transport in PHID: (**a**) Electrical potential distribution in the hydrogel heterojunction for various ionic strengths, no external electric field is applied; (**b**) conductivity profile in the hydrogel junction at different external bias polarities at the ionic strength of 100 μM; (**c**,**d**) plots of ionic conductivity along the center z-axis (AD in Figure S1) for various ionic strengths under a (**c**) forward bias and (**d**) reverse bias, respectively; (**e**) I-V characteristics of the entire hydrogel junction at various ionic strengths; (**f**) rectification ratios of the hydrogel junction obtained from respective I-V curves.

In addition to testing the device experimentally, we performed a numerical analysis to further study the ionic rectification characteristics. In the model, hydrogels are simplified to 2D nanopillar arrays (Figure S1, Supplementary Information) and the electrokinetic effect is neglected. Figure 3a displays the electrical potential distribution around the junction center with no external bias applied at three distinct ionic levels. Note that in all three conditions, the Debye length $\lambda_{D}$ (30.3–303.2 nm) is greater than the nanopore size (fixed at 20 nm). It can be seen that the potential profile in the nanoporous region does not align with that in the reservoirs and is rather dominated by the charged pillar surfaces as the electrical double layers overlap extensively. Interestingly, although the surface charge density is fixed (±2 mC/m^2^), the electrical potential on the pillar surfaces seems to decay with an increase in ionic strength. For 1, 10, and 100 μM KCl solutions, the maximum absolute value of potentials on the pillar surfaces are 0.186, 0.126, and 0.066 V, respectively. Under such a condition, the electrical double layers around a single pillar interact intensively with the double layers of surrounding ones which may affect the electrical potential on pillar surfaces when equilibrium is reached. This may help explain the increasing conductance of nanochannels under extremely low ionic strengths experimentally observed in a previous work [24]. Moreover, it should be noted that this tendency could be specific to the condition where $\lambda_{D}$ is greater than the critical length of the geometry. Indeed, an opposite trend was found in a previous numerical study, in which the potential on the surfaces of a conical nanopore was boosted by increasing the ionic strength under the condition that $\lambda_{D}$ was smaller than the pore size [14].

Next, we applied external potentials to the junction to monitor its ICR behavior. Figure 3b shows the numerically obtained conductivity profile in the junction under different bias polarities at an ionic strength of 100 μM. It can be observed that at a *V*_ap_ of 1 V, the conductivity is significantly increased, especially around the P-N gel interface, owing to the localized enrichment of ions. In this scenario, the ionic diode experiences a forward bias. At a *V*_ap_ of −1 V, however, ions are depleted from the P-N gel interface and the conductivity is much lower than the bulk value, leading to a reverse bias in which the ionic diode prevents the ionic current from flowing through. To visualize the relationship between conductivity and ionic strength, we plotted the ionic conductivity along the center z-axis AD (Figure S1) under different bias conditions. As shown in Figure 3c, under forward biases, the conductivity values in the junction area are markedly lifted over the bulk. Moreover, the values are positively correlated to the ionic strength. Under reverse biases, however, the conductivity values in the junction are rather independent of ionic strength except for the depletion zone near the P-N gel interface. In the depletion zone, the conductivity can be as low as ~$6.5\times 10^{-4}$, $6.2\times 10^{-6}$, and $5.8\times 10^{-8}$ mS/m for the ionic strengths of 100, 10, and 1 μM, respectively. The conductivity profile of the hydrogel junction is closely related to its overall voltage-current response, as shown in Figure 3e. As can be seen in Figure 3f, R values do not exhibit a noticeable tendency, which is in strong agreement with the experimental results (Figure 2a). These results suggest that the ICR behavior may not be directly dependent on ionic strength when $\lambda_{D}$ is greater than the critical dimension. Instead, one may consider the trade-off between $\lambda_{D}$ and pore size. In fact, the saturation of R was also observed in previous experimental studies of nanopores, which was explained by the saturation of counterions governed by the charged surfaces [14,25]. Although the simulated results qualitatively agree with the experiments, it is worthwhile noting that the quantitative R values are not strictly aligned. The discrepancy, here, can arise from leaking currents in the experiments which might lead to enlarged current especially at reverse biases and errors in the modeling process such as inaccurate modeling of hydrogel porous structure.

**Figure 4:**
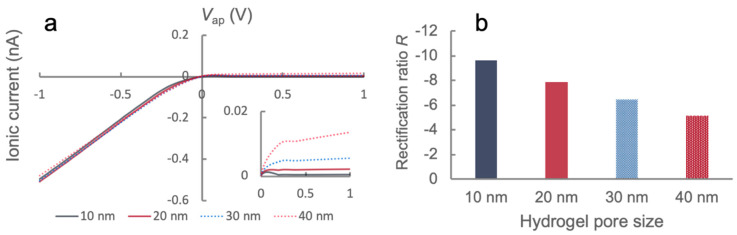
Simulation of the effects of hydrogel pore size on ionic transport characteristics in PHIDs: (**a**) I-V characteristics of the hydrogel junction for various pore sizes. The inset highlights the ionic currents when *V*_ap_ > 0; (**b**) Rectification ratios of the hydrogel junction obtained from respective I-V curves.

Next, we examined the effect of nanopore size on the ionic transport characteristics of the PHID. In the process, we fixed the ionic strength to 100 μM and the pillar surface charge density to ±2 mC/m^2^. Under such a condition, the potential profile in the junction was more or less maintained (data not shown). Therefore, we directly jumped to inspect the ionic transport characteristic. Figure 4 plots the I-V curves and R values for varying pore sizes. It can be seen that the ionic currents at forward biases (Figure 4a) were almost identical and independent of pore size, which was also observed in our previous experiments (Figure 2b). However, the current at reverse biases are significantly different (inset), which leads to considerable changes in the rectification ratio (Figure 4b). The change of rectification ratio strictly follows the trend that R decreases with an increase in pore size, which aligns with the experimental results in Figure 2b. The trend can be explained by the intensified electrical double layer overlapping when reducing the pore size, which leads to severer depletion around the P-N gel interface at reverse biases.

**Figure 5:**
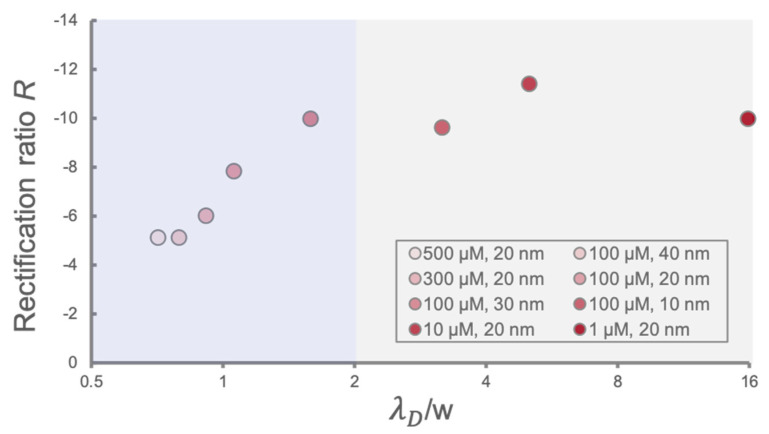
Plot of rectification ratios numerically obtained at various $\lambda_{D}/w$ values ($\lambda_{D}/w$ is displayed in logarithmic scale). The legend denotes the respective ionic strength and pore size condition for each data point.

With extensive discussions about the effect of ionic strength and pore size on the ion transport behavior of a PHID, we continued to ask whether ICR could be described by a simple dimensionless number. The ionic strength term can be represented by the characteristic length of electrical double layers, $\lambda_{D}$, using Equation (3). As we considered, the ratio between $\lambda_{D}$ and the pore size w could be a promising candidate to monitor the overall rectification behavior, because it reflects the degree of double-layer overlapping in nanopores, and thus the degree of surface-governed ion transport. In fact, existing experiments have shown that ICR increases with increasing $\lambda_{D}/w$ to a certain extent, for the case of conical nanopores [25]. To explore whether the trend could be numerically obtained, we plotted the rectification ratio R against $\lambda_{D}/w$ for various ionic strengths and pore sizes, as shown in Figure 5. Excitingly, we found that R indeed increased with $\lambda_{D}/w$, when $\lambda_{D}$ was comparable to w (0.5–2 for $\lambda_{D}/w$, highlighted by the blue background color in Figure 5), which partially verified our hypothesis that ICR can be characterized by the degree of double layer if overlapped in a moderate range. When $\lambda_{D}$ is considerably higher than w, however, the trend no longer exists (highlighted by grey background color), and R seems to reach a saturation stage. This could be explained, in part, by the ion saturation effect, i.e., with a decrease in the ionic strength, the electrical double layers nearly occupy the entire nanopore. In this case, the conductivity solely relies on the transport of counterions which is governed by the surface charge. As a result, further expanding the electrical double layers would not possess significant changes on the ICR since it is saturated. Further efforts are required to resolve the ICR characteristics at the saturation region.

**Figure 6:**
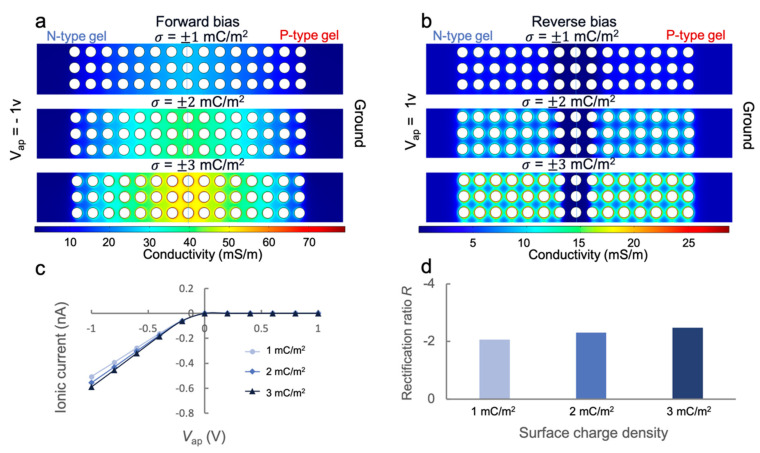
Simulation of the effect of surface charge density (σ) on the ion transport in the PHID: (**a**,**b**) Conductivity profile in the hydrogel junction for different surface charge density values at forward (**a**) and reverse (**b**) biases; (**c**) I-V characteristics of the hydrogel junction for various amplitudes of σ; (**d**) rectification ratios of the hydrogel junction obtained from respective I-V curves.

After understanding the role of ionic strength and pore size on rectification, next, we investigated the effect of surface charge density of the polyelectrolyte backbone on ICR behavior. This is important because it can predict whether the PHID device can be practically used to detect biomolecules (e.g., DNA and protein) based on their intrinsic charge. Here, to simulate the process of negatively charged DNA molecules captured within N-type gel, we fixed the charge density (σ) of the P-type gel and varied that of the N-type gel from 1 to 3 mC/m^2^. Similar surface charge densities have been frequently used in previous studies of ICR [24]. The ionic strength was fixed to 100 μM with the fixed pore size of 20 nm. First, we increased the amplitude of σ and observed increased conductivities in both forward (Figure 6a) and reverse biases (Figure 6b). Importantly, the conductivities increased most significantly around the pillar surfaces, especially noticeable at reverse biases. This suggests that one can expect a reasonable surface current effect with higher surface charges, which may lead to enhanced rectification. Indeed, both the ionic currents and the rectification ratio experienced a noticeable increase with higher σ values, as shown in Figure 6c,d. These results predict that the ICR of our PHID device is sensitive to surface charge and can be potentially applied to charge-based biosensing.

### 3.3. Detection of DNA with the PHID Device

**Figure 7:**
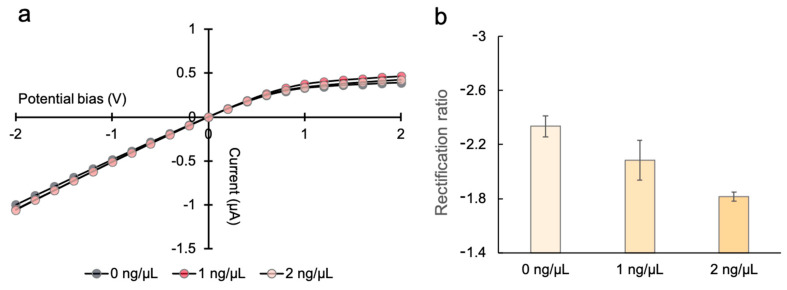
Electrical detection of DNA with the PHID device: (**a**) I-V curves; (**b**) corresponding rectification ratios (right, n = 3) of the representative PHID device subject to various DNA concentrations. The ionic currents were experimentally recorded at an ionic strength of 10 mM KCl.

With the insights gained from the simulation results, we experimentally tested our PHID device to determine if it could respond to the presence of DNA molecules in the environment. In the experiments, the N-type gel side of the hydrogel heterojunction was exposed to a DNA solution (500 bp double-stranded DNA). The DNA molecules in the solution were expected to be immobilized on the N-type gel backbone due to electrostatic interactions, and hence change the surface charge. As shown, the I-V characteristic was shifted slightly from its original state (0 ng/μL) upon exposure to DNA (Figure 7a). The rectification ratio decreased by 0.25 and 0.52, respectively, for 1 and 2 ng/μL DNA (Figure 7b). Such an exhibited trend is in qualitative agreement with the simulation results in Figure 6d. The results verify that the DNA molecules do interact with the N-type gel surfaces and, importantly, such electrostatic interactions are prominent enough to modulate the overall ionic current behavior of the device. Moreover, the results show that the change in *R* is relevant to DNA concentration, suggesting that the device can be applied to quantitatively detect DNA with a sensitivity of <1 ng/μL.

It should be noted that the PHID device presented here lacks sequence specificity to DNA molecules. That means the device detects the mass concentration of DNAs regardless of their base pair information. In fact, it should indistinctively detect any (negatively) charged macromolecules. An ideal application scenario for the device would be monitoring the amplification of nucleic acids where only specific product can be obtained. Whether the device is sensitive to the length of DNAs is yet to be explored. A smaller geometry of the hydrogel heterojunction would lead to enhanced sensitivity. Compared to other ionic diodes (e.g., nanopipettes), PHID devices are fully integrated, and thus more robust and amenable to fluidic integration and automation. Therefore, we anticipate that such novel ionic diode devices would offer interesting possibilities in the field of biosensing.

In conclusion, we have performed a systematic characterization of a polyelectrolyte hydrogel ionic diode, both experimentally and numerically. Our study has dissected major physical quantities that affect ionic rectification behavior. According to the results of charge sensitivity and field effect, the rectification has been verified to be responsive to charged biomolecules. Current efforts are underway to develop PHID-based biosensors for sensitive and multiplexed detection of important biomarkers.

## Supplementary Materials

The following are available online at https://www.mdpi.com/article/10.3390/s21248279/s1, Figure S1: Schematic illustration of the symmetric 2D computational domain, Table S1: Boundary conditions for the 2D axial symmetric computational domain in Figure S1.

## References

[1] Billiet T., Vandenhaute M., Schelfhout J., Van Vlierberghe S., Dubruel P. (2012). A review of trends and limitations in hydrogel-rapid prototyping for tissue engineering. Biomaterials.

[2] Mandal A., Clegg J.R., Anselmo A.C., Mitragotri S. (2020). Hydrogels in the clinic. Bioeng. Transl. Med..

[3] Le Goff G.C., Srinivas R.L., Hill W.A., Doyle P.S. (2015). Hydrogel microparticles for biosensing. Eur. Polym. J..

[4] Ma C., Li J., Zhang B., Liu C., Zhang J., Liu Y. (2019). Hydrogel Microparticles Functionalized with Engineered *Escherichia coli* as Living Lactam Biosensors. Sensors.

[5] Tang T.-C., Tham E., Liu X., Yehl K., Rovner A.J., Yuk H., de la Fuente-Nunez C., Isaacs F.J., Zhao X., Lu T.K. (2021). Hydrogel-based biocontainment of bacteria for continuous sensing and computation. Nat. Chem. Biol..

[6] Chun H., Chung T.D. (2015). Iontronics. Annu. Rev. Anal. Chem..

[7] Lee H.-R., Woo J., Han S.H., Lim S.-M., Lim S., Kang Y.-W., Song W.J., Park J.-M., Chung T.D., Joo Y.-C. (2019). A Stretchable Ionic Diode from Copolyelectrolyte Hydrogels with Methacrylated Polysaccharides. Adv. Funct. Mater..

[8] Putra B.R., Aaronson B.D., Madrid E., Mathwig K., Carta M., Malpass-Evans R., McKeown N.B., Marken F. (2017). Ionic Diode Characteristics at a Polymer of Intrinsic Microporosity (PIM)|Nafion “Heterojunction” Deposit on a Microhole Poly(ethylene-terephthalate) Substrate. Electroanalysis.

[9] Yin M.-J., Yin Z., Zhang Y., Zheng Q., Zhang A.P. (2019). Micropatterned elastic ionic polyacrylamide hydrogel for low-voltage capacitive and organic thin-film transistor pressure sensors. Nano Energy.

[10] Lim S.-M., Yoo H., Oh M.-A., Han S.H., Lee H.-R., Chung T.D., Joo Y.-C., Sun J.-Y. (2019). Ion-to-ion amplification through an open-junction ionic diode. Proc. Natl. Acad. Sci. USA.

[11] Lei Z., Zhu W., Zhang X., Wang X., Wu P. (2021). Bio-Inspired Ionic Skin for Theranostics. Adv. Funct. Mater..

[12] Cayre O.J., Chang S.T., Velev O.D. (2007). Polyelectrolyte Diode: Nonlinear Current Response of a Junction between Aqueous Ionic Gels. J. Am. Chem. Soc..

[13] Cheng L.-J., Guo L.J. (2010). Nanofluidic diodes. Chem. Soc. Rev..

[14] Kubeil C., Bund A. (2011). The Role of Nanopore Geometry for the Rectification of Ionic Currents. J. Phys. Chem. C.

[15] Siwy Z.S. (2006). Ion-Current Rectification in Nanopores and Nanotubes with Broken Symmetry. Adv. Funct. Mater..

[16] Pérez-Mitta G., Peinetti A.S., Cortez M.L., Toimil-Molares M.E., Trautmann C., Azzaroni O. (2018). Highly Sensitive Biosensing with Solid-State Nanopores Displaying Enzymatically Reconfigurable Rectification Properties. Nano Lett..

[17] Liu Y., Yobas L. (2013). Label-free electrical quantification of amplified nucleic acids through nanofluidic diodes. Biosens. Bioelectron..

[18] Liu Y., Yobas L. (2014). Label-Free Specific Detection of Femtomolar Cardiac Troponin Using an Integrated Nanoslit Array Fluidic Diode. Nano Lett..

[19] Perry J.M., Zhou K., Harms Z., Jacobson S.C. (2010). Ion Transport in Nanofluidic Funnels. ACS Nano.

[20] Han J.-H., Kim K.B., Kim H.C., Chung T.D. (2009). Ionic Circuits Based on Polyelectrolyte Diodes on a Microchip. Angew. Chem. Int. Ed..

[21] Yang C., Suo Z. (2018). Hydrogel ionotronics. Nat. Rev. Mater..

[22] Bao B., Hao J.R., Bian X.J., Zhu X.B., Xiao K., Liao J.W., Zhou J.J., Zhou Y.H., Jiang L. (2017). 3d Porous Hydrogel/Conducting Polymer Heterogeneous Membranes with Electro-/Ph-Modulated Ionic Rectification. Adv. Mater..

[23] Wang J.-Y., Xu Z., Li Y.-K., Liu C., Liu J.-S., Chen L., Du L.-Q., Wang L.-D. (2013). Nanopore density effect of polyacrylamide gel plug on electrokinetic ion enrichment in a micro-nanofluidic chip. Appl. Phys. Lett..

[24] Liu Y., Yobas L. (2016). Slowing DNA Translocation in a Nanofluidic Field-Effect Transistor. ACS Nano.

[25] White R.J., Zhang B., Daniel S., Tang J., Ervin E.N., Cremer P.S., White H.S. (2006). Ionic Conductivity of the Aqueous Layer Separating a Lipid Bilayer Membrane and a Glass Support. Langmuir.

