# Supplementary material for: An Experimental and Numerical Study of Polyelectrolyte Hydrogel Ionic Diodes: Towards Electrical Detection of Charged Biomolecules
Source: Sensors (Basel). 2021 Dec 10;21(24):8279. doi: 10.3390/s21248279 (PMC8707621; doi:10.3390/s21248279)
Supplement: Supplementary file 1 [file sensors-21-08279-s001.zip › sensors-1466038-supplementary.pdf]

Experimental and numerical study of polyelectrolyte hydrogel ionic diodes: towards electrical detection of charged biomolecules

Supplementary Information

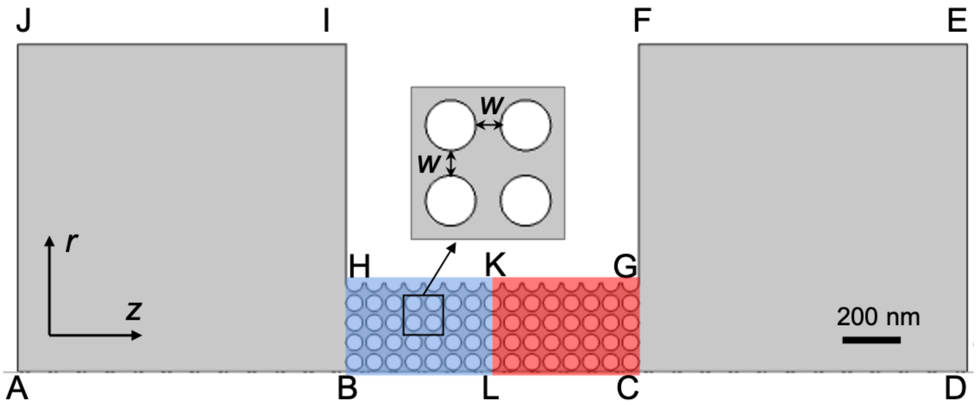

Figure S1. Schematic illustration of the symmetric 2D computational domain (axial symemtry along AD). The physical conditions for each boundary are detailed in Table 1. The blue and red colored regions depict the polyelectrolyte heterojunction.

Table S1. Boundary conditions for the 2D axial symmetric computational domain in Fig. S1.

Boundary	Length	Poisson	Nernst-Planck
AD	2.9 μm	axial symmetry	axial symmetry
DE	1 μm	constant potential (Ground)	constant concentration
AJ	1 μm	constant potential (V_{ap})	constant concentration
HK	0.45 μm	positive surface charge	insulation
KG	0.45 μm	negative surface charge	insulation
IJ, FE	1 μm	zero charge	insulation
HI, FG	0.73 μm	zero charge	insulation
Pillar surfaces in BLKH		positive surface charge	insulation
Pillars surfaces in CGKL		negative surface charge	insulation
